# Surface Electromyography Spectral Parameters for the Study of Muscle Fatigue in Swimming

**DOI:** 10.3389/fspor.2021.644765

**Published:** 2021-02-19

**Authors:** Luca Puce, Ilaria Pallecchi, Lucio Marinelli, Laura Mori, Marco Bove, Daniele Diotti, Piero Ruggeri, Emanuela Faelli, Filippo Cotellessa, Carlo Trompetto

**Affiliations:** ^1^Department of Neuroscience, Rehabilitation, Ophthalmology, Genetics, Maternal and Child Health, University of Genoa, Genoa, Italy; ^2^National Research Council (CNR), SPIN institute, Department of Physics, Genoa, Italy; ^3^Department of Neuroscience, IRCCS Ospedale Policlinico San Martino, Genoa, Italy; ^4^Department of Experimental Medicine, Section of Human Physiology, University of Genoa, Genoa, Italy; ^5^Centro Polifunzionale di Scienze Motorie, University of Genoa, Genoa, Italy

**Keywords:** electromyography, spectral parameters, fatigue, swimming, master swimmers, video analysis

## Abstract

The purpose of this study was to assess validity, stability and sensitivity, of 4 spectral parameters–median frequency (F_med_), mean frequency (F_mean_), Dimitrov index (DI), and mean instant frequency (F_mi_)–in measuring localized muscle fatigue in swimming and to investigate their correlation with the variations of kinematic data and mechanical fatigue. Electrophysiological measures of muscle fatigue were obtained in real-time during a 100 m front crawl test at maximum speed in 15 experienced swimmers, using surface electromyography in six muscles employed in front crawl, while kinematic data of swimming was measured from video analysis. Mechanical fatigue was measured as the difference between muscle strength prior to and immediately after the 100 m front crawl in a dry-land multi-stage isometric contraction test. Statistically significant fatigue (*p* < 0.0001) was found for all spectral parameters in all muscles. F_med_ and F_mean_ varied between 10 and 25%, DI between 50 and 150%, and F_mi_ between 5 and 10%. Strong correlation (Pearson *r* ≥ 0.5) with mechanical fatigue was found for all spectral parameters except for F_mi_ and it was strongest for F_med_ and F_mean_. From our study, it turns out that F_med_ and F_mean_ are more valid and stable parameters to measure fatigue in swimming, while DI is more sensitive.

## Introduction

Fatigue has been defined as “*a reduction in force output that occurs during sustained voluntary activity”* (Bigland-Ritchie et al., 1983), and more recently as “*any exercise-induced loss of ability to produce force with a muscle or muscle group”* (Taylor et al., 2006). The phenomenon of fatigue is a common experience in sports, particularly complex as it varies with the change in the type of exercise performed. In particular swimming is a dynamic task and it requires coordinated activation of lower limbs, core, and upper body muscles in each stroke cycle. In addition, the water environment does not offer a fixed fulcrum to exert a maximal force, indeed muscle force at each pulling stroke is only about 50% of the maximal voluntary contraction (Stirn et al., 2011). The decay of velocity and the variations of the kinematic parameters are widely used methods to monitor fatigue in swimming (Stirn et al., 2011; Ikuta et al., 2012; Figueiredo et al., 2013; Conceição et al., 2014; Puce et al., 2018). Although relatively simple to determine, these methods are neither direct nor muscle-specific measurements of fatigue. Real-time monitoring of localized muscle fatigue during the execution of a task is possible through surface electromyography (EMG). During a prolonged muscle contraction, as consequence of the physiological mechanisms of fatigue, the spectral weight of the EMG shifts from high to low frequencies (Dimitrov et al., 2006; González-Izal et al., 2012). For this reason, the time evolution of power spectrum parameters such as the median frequency (F_med_), the mean frequency (F_mean_), the Dimitrov index (DI) (Dimitrov et al., 2006) and the mean instant frequency (F_mi_) could be used to detect the electrophysiological signs of localized muscle fatigue in swimming. Two studies monitored the variation of F_mean_ (Stirn et al., 2011; Conceição et al., 2014). Stirn et al. reported that at the end of a 100 m front crawl, F_mean_ decreased significantly by 20–25% in the upper body muscles. In the study of Conceição et al., despite the evolution of kinematic and physiological parameters reflected the development of muscle fatigue during 200 m breaststroke, only a non-significant trend of F_mean_ decrease in the upper body muscles was reported. Front crawl and breaststroke are technically different in terms of functional involvement of muscles, and consequently fatigue. The upper body muscles, monitored in both studies, are more active in front crawl than in breaststroke, as in breaststroke most of the propulsion is provided by lower body muscles. For this reason, a direct comparison of these apparently conflicting results is not possible.

In order to investigate the evolution of fatigue in a 200-m front crawl, Figueiredo et al. (2013) used DI, which is thought to be more sensitive than F_med_ and F_mean_ as a measure of fatigue in sub-maximal contractions (Dimitrov et al., 2006; González-Izal et al., 2012). The results of this work showed a significant increase of DI by 40–60% for upper limb muscles, but no significant variation for those in lower limbs. Spectral analyses of EMG signal that rely on the Fourier transform (F_med_, F_mean_ and DI), are based on the assumption that the signal is stationary during the analyzed 0.5–2.0 s intervals (Cifrek et al., 2009). This may not be the case for EMG signals associated to dynamic contractions, especially involving fast movements, where myoelectric signal bursts are often shorter than 500 ms (Bonato et al., 1996). On the other hand, alternative analysis models have been used for non-stationary EMG signals, such as the short-time Fourier transformation (STFT), where the FFT is applied to short overlapping stationary intervals, to the detriment of frequency resolution, the autoregressive or autoregressive–moving-average methods (Witte et al., 2006), the Wavelet methods based on intensity analysis, the methods based on transforms that work well for non-stationary and non-linear data. Among other time-frequency distributions that do not require the hypothesis of stationarity of the EMG signal, are the Cohen class time-frequency transforms (Cifrek et al., 2009), which may thus be more suitable for the spectral analysis in the case of dynamic contractions (Bonato et al., 1996, 2001; González-Izal et al., 2010). Caty et al. (2006) calculated F_mi_ using the Choi-Williams transform, belonging to the Cohen's class transforms, in a 4 × 50 m front crawl and observed a decrease in F_mi_ for the *extensor carpi ulnaris* and *flexor carpi ulnaris* muscles by 11 and 9%, respectively. These variations of F_mi_ were sizably smaller than the variations of spectral parameters based on the Fourier transform (Stirn et al., 2011; Figueiredo et al., 2013; Conceição et al., 2014). From the above mentioned reports, it appears that further studies are necessary to gain insight on the validity of the methods of spectral analysis of EMG signal, for the assessment of localized muscle fatigue in swimming. Yet, valid, stable and sensitive methods to measure fatigue could be useful to assess the level of performance, prevent injuries (Matthews et al., 2017) and adjust training methods (Puce et al., 2018). In this work, we present the evolution of the 4 spectral parameters F_med_, F_mean_, DI and F_mi_ during a 100 m front crawl and their correlation with the variations of the kinematic data and the peak torque. Our final aim is to assess validity, stability, and sensitivity of each spectral parameter in measuring the localized muscle fatigue in swimming.

## Methods

### Subjects

Fifteen elite masters swimmers (two women; mean ± standard deviation age 33.0 ± 9.7 years; weight 71.9 ± 9.5; height 177.8 ± 8.6 cm) took part in the research study, after 10 days of tapering phase. The swimmers were front crawl specialists, even if some of them were not sprinters. Their average technical index was considered high (612 ± 43) (Santos et al., 2020).

The study was carried out in accordance with the code of ethics of the World Medical Association (Declaration of Helsinki 2014) for experiments involving humans. A written informed consent was obtained from all participants prior to participation in the study. The project was approved by the local ethics committee (University of Genova, Italy. N. 2020/21).

### Study Design

Electrophysiological measures of muscle fatigue and kinematic data were obtained in real-time during a 100 m front crawl, using EMG and video analysis. Mechanical fatigue was measured as the difference between muscle strength prior to and immediately after the 100 m front crawl in a dry-land multi-stage Isometric Contraction Test (MICT). After the MICT performed at rest (pre-MICT), a 30 min time was used for recovery and for application of EMG electrodes and adhesive markers. Then the 100 m front crawl Swimming Fatigue Test (SFT) was carried out. The second MICT (post-MICT) was performed immediately after the SFT. It must be stressed that the time elapsed between the end of the SFT and the post-MICT was kept at minimum (<10 s), in such a way that the level of muscular strength measured in the post-MICT was representative of the fatigue experienced in the SFT.

The outcome measures of this study were the variations of the four spectral parameters of the EMG signal, F_med_, F_mean_, DI and F_mi_, measured during the SFT, the variations of the peak torque measured before and after the SFT and the variations of the kinematics data, velocity, stroke length, and stroke index, measured during the SFT.

Validity of the spectral parameters was determined on the basis of the correlation of electrophysiological signs of fatigue with mechanical fatigue and kinematic parameters. Stability of the spectral parameters was estimated in terms standard deviation of data within individual 100 m SFTs. Sensitivity of the spectral parameter was determined in terms of range of variation in the SFTs.

### Swimming Fatigue Test (SFT)

Measurements were performed in a 50-m indoor swimming pool. After the pre-MICT and 30 min recovery, the swimmers performed an individual warm-up. After the warm-up, the swimmers were instructed to perform a 100 m front crawl at the highest level of self-perceived exertion. Due to the measuring equipment attached to the body, the underwater turn was allowed but the dive start was not.

### Multi-Stage Isometric Contraction Test (MICT)

**Figure 1:**
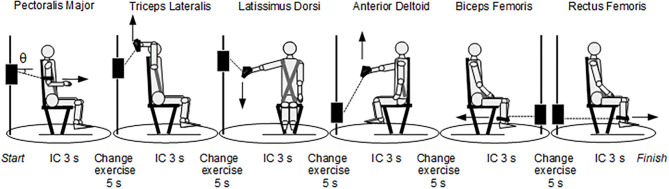
Multi-stage isometric contraction test (MICT). In the left-most position, θ indicated the angle between the cable and the horizontal axis. The joint and cable angles in the six positions are: Pectoralis Major: shoulder flexed in neutral position (0°) and elbow flexed by 90°, θ = −15°; Triceps Lateralis: shoulder flexed by 180° and elbow flexed by 90°, θ = +55°; Latissimus Dorsi: shoulder abducted by 75 °, θ = −45°; Anterior Deltoid: shoulder flexed by 75°, θ = +60°; Biceps Femoris: knee flexed by 90°, θ = 0°; Rectus Femoris: knee flexed by 90°, θ = 0°. IC = isometric contraction.

MICT had a total duration of 43 s and was carried out on the pool deck. It consisted of six isometric contractions lasting 3 s interspersed with 5 s (change of exercise), each one involving different muscles, carried out using a cable cross over apparatus (model Technogym Cable Stations Ercolina Rehab, Cesena, Italy) with a load cell connected to a Digital Force Indicator display (model C2S-AMP, Modena, Italy). For the pre-MICT and post-MICT tests to be equivalent, participants were asked to express strength at their maximum in the contractions. It must be pointed out that these cannot be considered as maximum voluntary contractions (MVC), which are commonly used in fatigue experiments, due to the short duration (3 s) of the effort. Yet, this duration was chosen to minimize the difference in recovery times among the six successive contractions in the post-MICT tests, as well as to avoid an overall fatigued state that developed in the participants through the MICT tests, in case of contraction durations of 5 s or longer. The maximum value that was maintained on the display for a time at least of the order of 1 s was considered as the peak force value. This force value was converted into a torque (Nm) for each exercise and the difference between pre-MICT and post-MICT torques was normalized to the pre-MICT torque value. The swimmer was harnessed to a chair and changed their body position for each contraction in such a way as to be biomechanically able to recruit a specific muscle (Figure 1). The first muscle used was the *pectoralis major* (PM), followed by the *triceps lateralis* (TL), *latissimus dorsi* (LD), *anterior deltoid* (AD), *biceps femoris* (BF) and *rectus femoris* (RF). The succession was structured to optimize the timing. The athletes carried out a pre-test warm-up and a month-long training to learn to compensate as little as possible with other muscles, keep a correct standard position, stay within the contraction/exercise change times, and express the maximum strength during the test.

### EMG Data Collection

EMG signals from PM, TL, LD, AD, BF, and RF of the dominant side were measured through bipolar surface electrodes using a waterproof wireless EMG equipment (Cometa srl, Milan, Italy) operating at 2,000 Hz, according to SENIAM guidelines (Hermens et al., 2000). To avoid alterations induced by underwater recording, a water resistant adhesive tape over the electrodes was applied (Rainoldi et al., 2004). The above six muscles were selected according to their relevance in front crawl (Clarys, 1983) and were the same as those assessed in the MICT. Data analysis was performed using the open source software Python distributed by Anaconda Inc. In each EMG trace, the activation interval [t_in_, t_fin_] of each stroke was identified where the envelope of the rectified signal around the maximum amplitude exceeded 20% of the maximum amplitude itself, following the same criterion of Stirn et al. (2011). Once the starting and ending times of the activation interval of each stroke were identified this way, spectral analysis was carried out on each activation interval. The EMG intervals were filtered with a band-pass Butterworth filter of 4-th order in the range of 20–500 Hz and then analyzed in the frequency domain. The four spectral parameters F_med_, Fmean, DI and Fmi were calculated.

F_med_ of the power spectrum was calculated as the frequency that divides the power spectrum into two parts having the same spectral weight, according to the following equations:

(1) $$\int_{f_{c1}}^{F_{med}}PSD\;(f)\;df=\int_{F_{med}}^{f_{c2}}PSD\left(f\right)df=\frac{1}{2}\int_{f_{c1}}^{f_{c2}}PSD\;(f)\;df$$

where f_c1_= 20 Hz and f_c2_= 500 Hz are the cut-off frequencies of the high-pass and low-pass filters applied to the spectra and PSD(f) is the power spectral density.

F_mean_ of the power spectrum was calculated as the momentum of order 1 of the power spectrum:

(2) $$Fmean=\frac{\int_{f_{c1}}^{f_{c2}}f\;\cdot PSD\;\left(f\right)\;df}{\int_{f_{c1}}^{f_{c2}}PSD\;\left(f\right)\;df}$$

DI was calculated as the ratio of the momentum of order −1 of the power spectrum to the momentum of order 5:

(3) $$DI=\frac{\int_{f_{c1}}^{f_{c2}}\frac{1}{f}\;\cdot PSD\;\left(f\right)\;df}{\int_{f_{c1}}^{f_{c2}}f^{5}\cdot PSD\;\left(f\right)\;df}$$

The instant frequency IF(t) was calculated using the Choi–Williams time-frequency distribution, with intermediate value of the kernel parameter (O'Toole and Boashash, 2013). F_mi_ was then calculated by averaging IF(t) over each activation interval between times t_in_ and t_fin_:

(4) $$IF\left(t\right)=\frac{\int_{f_{c1}}^{f_{c2}}f\;\cdot \;{\left|CW\left(t,f\right)\right|}^{2}df}{\int_{f_{c1}}^{f_{c2}}{\left|CW\left(t,f\right)\right|}^{2}df},\;Fmi=\frac{\int_{t_{in}}^{t_{fin}}IF\left(t\right)dt}{t_{fin}-t_{in}}$$

Here |CW(t,f)|^2^ are the squared time-frequency components of the Choi-Williams transform.

Electrophysiological detection of muscle fatigue were obtained as time variation of spectral index, quantified by the slope of the linear regressions of spectral index vs. time, with uncertainty given by the standard deviation of the linear regression. A negative slope for F_med_, F_mean_, F_mi_ and positive slope for DI indicates fatigue. The slopes were finally normalized to the initial value of the regression line for each EMG trace. Average values of normalized slopes of each spectral parameter for each muscle were calculated over the 15 participants. The average of each spectral parameter x over the participants was calculated by weighting each value with the inverse variance $\frac{1}{\sigma_{i}^{2}}$ obtained from the linear regression:

(5) $$x_{average}=\;\frac{\sum_{i=1}^{15}\frac{x_{i}}{\sigma_{i}^{2}}}{\sum_{i=1}^{15}\frac{1}{\sigma_{i}^{2}}}$$

The error bars on these averages were calculated as:

(6) $$\sigma_{x}=\;\sqrt{\frac{1}{\sum_{i=1}^{15}\frac{1}{\sigma_{i}^{2}}}}$$

### Kinematic Data Collection

The measurements of kinematic data were carried out by analyzing video recordings (Kinovea 0.8.25), acquired on sagittal plane using two cameras (model GoPro Hero 8, GoPro, San Mateo, CA, USA) one above the water surface and one below. The cameras were fixed to a pushcart which was moved at the same speed as the swimmer speed. Precise information on the absolute position of body and limbs was obtained by applying adhesive markers on the joints of the lower and upper limbs and synchronizing the biomechanical analysis with the EMG signal. Specifically, triggering of video recording and EMG signal was done by tapping a spare EMG probe at the start. Swimming velocity (SV), stroke length (SL), and stroke index (SI) were evaluated. SI was calculated as the product of SV and SL, and it was used as an index of the swimming efficiency (Costill et al., 1985). The SV was calculated as the ratio of space swum to chronometric time. The SL was calculated as the ratio of space swum to the corresponding number of strokes. To avoid the influence of the start and turn phases, all three parameters were calculated in the free-swimming segment, that is between 15th and 45th m of the pool length. Finally, the variations of SV, SL and SI were evaluated as the linear time derivatives $\left(\frac{dSI}{dt},\frac{dSV}{dt},\;\frac{dSL}{dt}\right)$ in the free swimming segments and normalized to the respective values at the instant t_0_ corresponding to the 15th m of the first length, *SI*(*t* = *t*_0_), *SV*(*t* = *t*_0_), *SL*(*t* = *t*_0_).

### Statistical Analysis

Correlation between electrophysiological signs of fatigue (normalized slopes of EMG spectral index) and mechanical fatigue (normalized difference in torque between pre-MICT and post-MICT) and between electrophysiological signs of fatigue and decay of kinematic parameters (normalized slopes of kinematic parameters) was evaluated by the Pearson coefficient r. It was assumed that correlation was *strong* for 0.5 ≤ |r| ≤ 1, *moderate* for 0.3 ≤ |r| ≤ 0.49, *low* for |r| ≤ 0.29, *null* for |r| = 0. One-way ANOVA test was used to evaluate the significance of differences of electrophysiological signs of fatigue between different muscles. Statistical significance was also evaluated in terms of *p*-value for the average normalized slope of each spectral parameter for each muscle and for the average variation of kinematic parameters. Statistical significance was set at *p* < 0.05.

## Results

### Electrophysiological Signs of Fatigue

**Figure 2:**
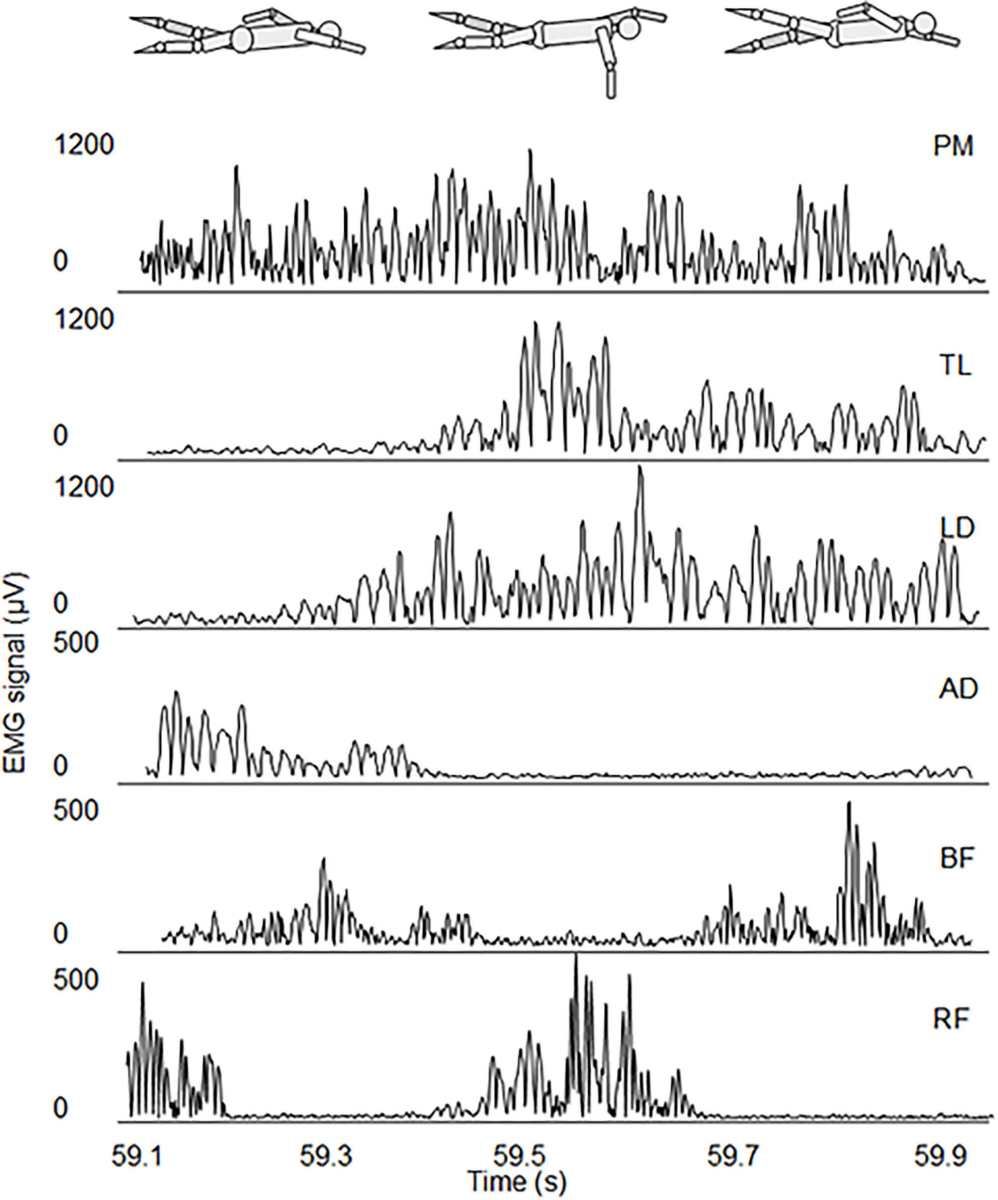
EMG activation of the underwater phase stroke of the 6 muscles of one representative participant during the SFT.

In Figure 2, we present a typical EMG acquisition during the underwater phase of a stroke, where the activation intervals of the 6 muscles and their relative shift are shown. The upper body muscles had one activation interval per stroke, while lower limbs have either two or three activation intervals per stroke, depending on the participant. PM has the longest activation interval.

**Figure 3:**
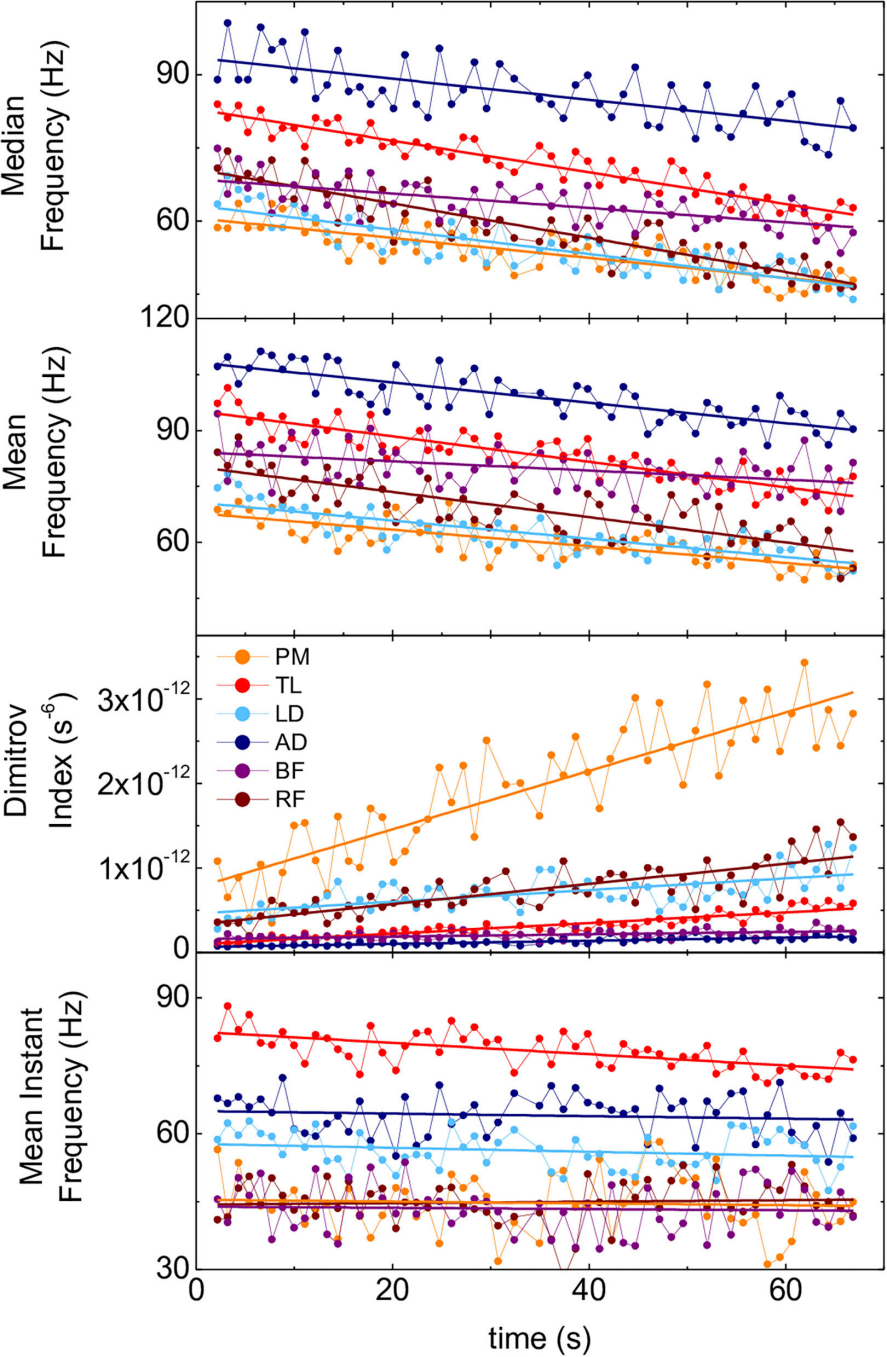
Time evolution of spectral parameters of the 6 muscles of one representative participant during the SFT. Panels from top to bottom: F_med_, F_mean_, DI, F_mi_. PM, pectoralis major; TL, triceps lateralis; LD, latissimus dorsi; AD, anterior deltoid; BF, biceps femoris; RF, rectus femoris.

F_med_, F_mean_, and F_mi_ for all the participants and muscles exhibited a decreasing trend over time while DI exhibited an increasing trend. Figure 3 shows recordings of a representative set of these parameters.

**Figure 4:**
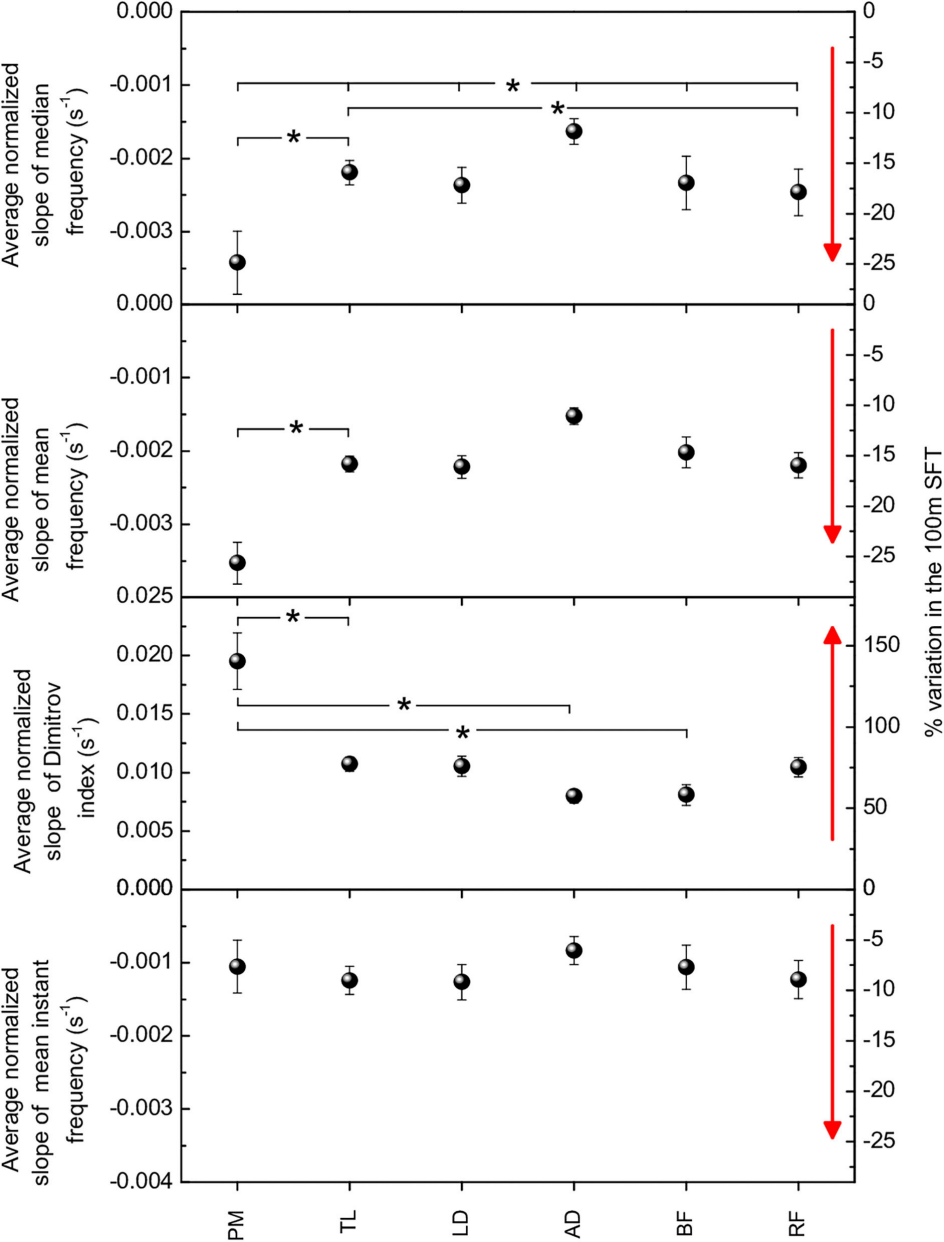
Weighted averages of the normalized slopes of spectral parameters for the six muscles. Panels from top to bottom: F_med_, F_mean_, DI, F_mi_. All these values are statistical significant (*p* < 0.0001). Right-hand axes indicate the percent variation in the FST. Asterisks indicate statistical significance (*p* < 0.05) of difference between either two or all six muscles. Red arrows point to the direction of increasing fatigue for each spectral parameter.

The average normalized slopes of each parameter and each muscle over the 15 participants are shown in Figure 4. All these values were statistically significant (*p* < 0.0001).

F_med_ and F_mean_ varied between 10 and 25%, DI between 50 and 150%, and F_mi_ between 5 and 10%. Regarding scattering of data for each parameter in individual SFTs (see sets of data in Figure 3 as an example), in absolute terms, the standard deviation of DI was on average seven times larger than the standard deviation of F_med_, 13 times larger than the standard deviation of F_mean_, and nine times larger than the standard deviation of F_mi_. In relative terms, normalizing to average values, the standard deviation of DI was still the largest (~70%), as compared to ~60% for F_med_ and F_mi_ and ~35% for F_mean_.

For all spectral parameters, larger relative changes among upper body muscles was observed in PM and LD, while the least relative change was observed in AD. In the lower limb muscles, the relative change was comparable for BF and RF, except in the case of DI, which showed larger variation for RF.

Regarding the differences between muscles, statistical significance (*p* < 0.05) was observed for the PM-TL and TL-RF couples and within the group of all the 6 muscles for F_med_; only for the PM-TL couple for F_mean_; for the PM-TL, PM-AD and PM-BF couples for DI.

### Mechanical Fatigue

**Figure 5:**
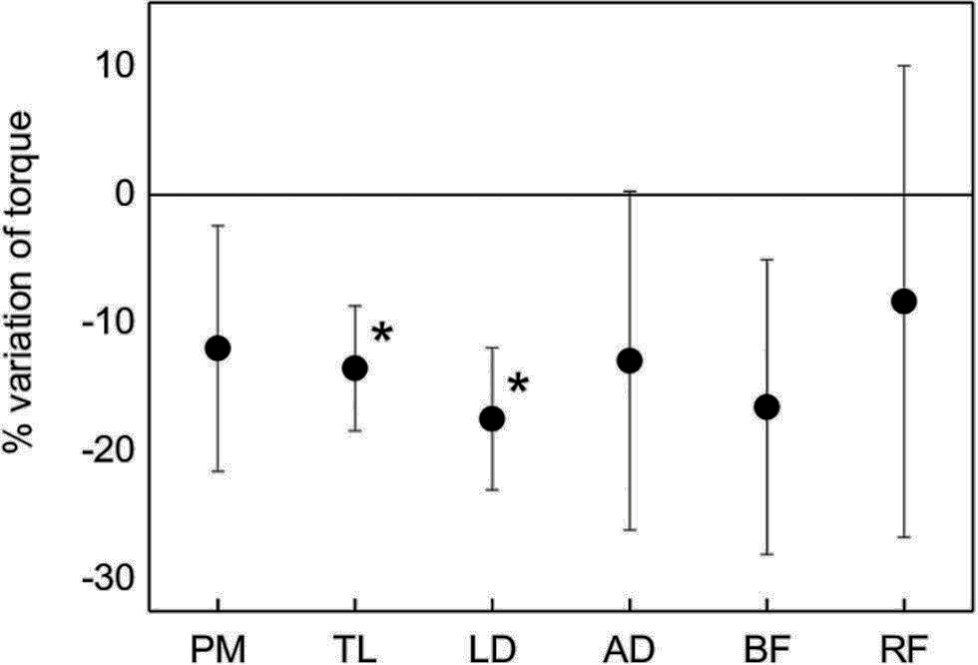
Average percent variation of the torque for each muscle from the pre-MICT to the post-MICT, representing mechanical fatigue. Asterisks indicate statistical significance (*p* < 0.05).

Mechanical fatigue, evaluated as normalized difference between post and pre MICT varied between 8% for RF and 17% for LD, as shown in Figure 5. Due to the scattering of data, statistical significance was found only for TL and LD (*p* < 0.05).

### Kinematic Data

**Figure 6:**
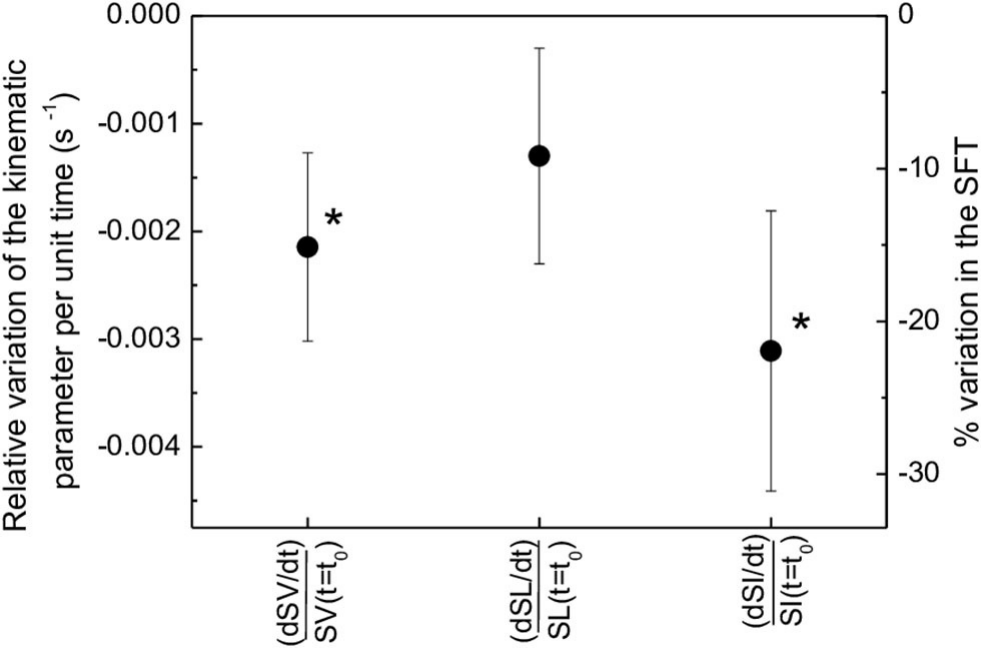
Average relative variation of the kinematic parameters (SV, SL, SI) per unit time in the FST, expressed as linear time derivative of each kinematic parameter ($\frac{dSI}{dt},\frac{dSV}{dt},\;\frac{dSL}{dt}$, respectively) normalized to the value of the kinematic parameter at the beginning of the free swimming segment [*SI*(*t* = *t*_0_), *SV*(*t* = *t*_0_), *SL*(*t* = *t*_0_), respectively]. Asterisks indicate statistical significance (*p* < 0.05).

The average swimming time achieved by the swimmers was longer than their personal best by as little as 3.2%. Hence, considering the fact that in SFT the participants did not perform the dive start, did not wear a racing suit and had to cope with the burden of the equipment, the performance can be considered performed at maximum effort. Moreover, for all participants a progressive decrease of SV, SL, and SI was observed. Figure 6 presents the normalized slope of the three kinematic parameters across the FST. SV, SL, and SI exhibited variations by 15, 9, and 22%, respectively. Statistical significance applied to SV and SI (*p* < 0.05).

### Correlation

For increasing fatigue F_med_, F_mean_, and F_mi_ should decrease and DI increase, and torque should decrease as well. In these conditions, all the characteristic spectral frequencies (F_med_, F_mean_, and F_mi_) correlate positively with the changes in torque, while DI correlates negatively with the changes in torque. Indeed, this is just what comes out from the Pearson coefficients r, reported in Table 1, used to evaluate the correlation between electrophysiological signs of fatigue, assessed through different spectral parameters, and mechanical fatigue.

**Table 1:** Pearson correlation coefficient r_Pearson_ coefficients between normalized variations of spectral parameters (electrophysiological fatigue) and normalized variations of torque (mechanical fatigue).

**Muscle**	**Spectral parameter**			
	**F_MED_**	**F_MEAN_**	**DI**	**F_MI_**
PM	0.61	0.59	−0.42	−0.37
TL	0.88^*^	0.83^*^	−0.63^*^	0.20
LD	0.59^*^	0.66^*^	−0.03	−0.60
AD	0.90^*^	0.95^*^	−0.53	−0.15
BF	0.75^*^	0.78^*^	−0.68^*^	0.26
RF	−0.26	−0.33	0.57	−0.53

*Asterisks indicate statistical significance of the correlation (p < 0.05)*.

The negative variations of F_med_ and F_mean_ exhibited *strong* positive correlation with mechanical fatigue for all the muscles, except RF. Statistical significance of these correlations was found for TD, LD, AD, and BF. On the contrary, the negative variations F_mi_ exhibited positive correlation with mechanical fatigue only for TL and BF, but this correlation was *low* and not significant.

The positive variations of DI exhibited *strong* negative correlation with mechanical fatigue for TD, AD, and BF, with statistical significance for TL and BF, while for PM only moderate *correlation* was found.

Not significant correlation was found between the changes in spectral parameters and the changes in all kinematic parameters SV, SL, and SI.

## Discussion

In this study, we presented the evolution of the four spectral parameters during 100 m front crawl and their correlation with the variation of the torque and kinematic data to assess the validity and sensitivity of each spectral parameter that measures fatigue in swimming. From our study, it turned out that F_med_ and F_mean_ are more stable and valid parameters to measure fatigue in swimming, while DI is more sensitive.

### Electrophysiological Signs of Fatigue

The relative difference in fatigue between different muscle is qualitatively similar for all the spectral parameters, in agreement with results of Dimitrov studies (Dimitrova et al., 2005; Dimitrov et al., 2006, 2008). The widest range of variation was observed for DI revealing about six times larger sensitivity to fatigue than F_med_ and F_mean_ and 15 times larger than F_mi_. In other studies, DI was found to be up to 150 times larger than F_med_ and F_mean_ during electrically evoked contractions (Dimitrova et al., 2005) and 50 times larger during voluntary isometric contractions (Dimitrov et al., 2008).

Larger sensitivity of DI is due to definition as the ratio of momentum of order −1 of the power spectrum to the momentum of order five, which better describes the shift of spectral weight from high to low frequencies with increasing fatigue, due to different mechanisms. The spectral moment of order (−1) emphasizes the increase in low and ultralow frequencies in the EMG spectrum due to increased negative after-potentials during fatigue. The spectral moment of order five emphasizes the effect of decreases in high frequencies, due to increments in the duration of the intracellular action potentials and decrements in the action potential propagation velocity (Dimitrov et al., 2006). As a counterpart of such larger sensitivity, DI showed the largest standard deviations of the data within individual 100 m SFTs, likely due to numerical instability, originating from the stochastic nature of the EMG signal and the DI definition itself in terms of higher-order momenta of the power spectrum.

Comparing fatigue in different muscles, the largest fatigue was observed for PM. Indeed, in studies that analyse the EMG amplitude normalized of MVC (Clarys, 1983; Pink et al., 1991), PM was observed to produce the most propulsive force in front crawl, together with LD and TB. Moreover, from our results, the duration of its EMG activity in the front crawl stroke was the longest among the investigated muscles, lasting throughout the whole underwater phase (Figure 2). This may be due to the fact that PM, in synergy with other respiratory muscles, is also responsible for inspiration. LD and TB showed large fatigue, as well. LD is known as the “*swimmers muscle*” due to the major role it plays in the successful completion of each of the swim styles (Laudner and Williams, 2013) and, together with TB, it is considered the key muscle in maintaining swimming speed in fatigued conditions (Ikuta et al., 2012). AD was the least fatigued upper body muscle. The contribution of this muscle to propulsion is limited (Figure 2), and its main function is bringing the shoulder over the head during the recovery phase of the stroke (Pink et al., 1991). Although lower limb muscles only contribute about 15% to propulsion in front crawl (Stirn et al., 2011), fatigue in BB and RF was similar to upper body muscles. In front crawl the biomechanics of lower limbs do not rely on a good support surface, as in the case of breaststroke, however they have twice or three times as many activations intervals as the upper body muscles in each stroke (Figure 2).

### Correlation

The electrophysiological manifestation of fatigue obtained as change in spectral parameters over time is associated with phenomena that occur in the muscle, prior to occurrence of mechanical fatigue. Indeed, the variations of these parameters reflect physiological phenomena that will only subsequently degrade the mechanical performance of the muscle. Therefore, it is interesting to inspect the correlation between the parameters that reflect electrophysiological signs of fatigue and parameters that identify mechanical fatigue.

An important finding of the present work was that F_med_ and F_mean_ showed the highest correlation between electrophysiological signs of fatigue and mechanical fatigue. Hence, these spectral parameters proved to be the most valid in dynamic contractions, whose bursts are often shorter than 500 ms (Figure 2), a time interval in which the problem of possible non-stationarity of the EMG signal may arise.

Regarding F_mi_ it exhibited the least correlation between electrophysiological and mechanical fatigue and the least sensitivity to fatigue in different muscles. It must be noted that we observed a negligible dependence of results on the choice of the Choi–Williams parameter over a wide range of values. This parameter suppresses the cross-terms in the frequency spectrum, i.e., terms originating from the product of different frequency components, which appear as a consequence of non-stationarity of the signal. The negligible influence of this parameter on the results indicates that the problem of non-stationarity is not relevant in our signal. In this situation, the use of the Choi-Williams distribution in place of the Fourier transform is not appropriate, as it causes a smoothening of the frequency spectrum, thus also altering the frequency components of the signal and not just the mixed terms.

We remark that the correlation between electrophysiological and mechanical fatigue observed in our study was not obvious a priori. Indeed, dynamic and static contractions have different patterns of neural activations (Cheng and Rice, 2005).

The kinematic parameters were not correlated with electrophysiological measures of fatigue, represented by any of the spectral parameters. This result can be explained by the unaware tendency of swimmers to maintain constant velocity in fatigued conditions, through modification of arm coordination (Cheng and Rice, 2005) and muscle activation (Stirn et al., 2011; Ikuta et al., 2012). This unaware compensation strategy may be a further reason why fatigue occurs differently for different muscles.

## Practical Implications

This study provides information on the use of the most appropriate spectral parameters in terms of validity, stability and sensitivity for the assessment of fatigue in swimming. Our results show that F_med_ and F_mean_ are the most valid and stable parameters and are thus recommended, particularly in tests where maximum effort is required. DI is the most sensitive, and it may be more suitable in tests where low intensity muscle contractions are required, although it is intrinsically more liable to numerical instability, due to the stochastic nature of the EMG signal.

## Limitations of This Study

We point out two limitations of this study. First, the positions of the MICT were optimized in neither singling out the effort of a specific muscle, nor in simulating the in-water stroke movement. However, the MICT provided a good measure of mechanical fatigue as long as it was performed in identical conditions prior to and after the swimming fatiguing test. A second limitation regards the measurement of the mechanical force, which was carried out by visual inspection of the dynamometer display, rather than by recording and mathematic averaging the output signal of this instrument. However, attention was paid to ensure reproducibility of the method.

## Data Availability Statement

The raw data supporting the conclusions of this article will be made available by the authors, without undue reservation.

## Ethics Statement

The studies involving human participants were reviewed and approved by the Local Ethics Committee (University of Genova, Italy. N. 2020/21). The participants provided their written informed consent to participate in this study. The patients/participants provided their written informed consent to participate in this study.

## Author Contributions

LP ideated and participated at the experiment and wrote the manuscript. CT ideated the experiment and wrote the manuscript. FC, DD, and LMa participated in the experiment and in the literature search. EF performed the EMG experiment and in the literature search. PR performed the EMG experiment and collaborated in revising the manuscript. MB ideated the experiment and collaborated in revising the paper. LMo and IP analyzed the data and collaborated in revising the manuscript. All authors read and approved the final version of the manuscript.

## Conflict of Interest

The authors declare that the research was conducted in the absence of any commercial or financial relationships that could be construed as a potential conflict of interest.
